# Syntactic Recursion Facilitates and Working Memory Predicts Recursive Theory of Mind

**DOI:** 10.1371/journal.pone.0169510

**Published:** 2017-01-10

**Authors:** Burcu Arslan, Annette Hohenberger, Rineke Verbrugge

**Affiliations:** 1 Institute of Artificial Intelligence, University of Groningen,AK Groningen, The Netherlands; 2 Department of Cognitive Science, Middle East Technical University, Üniversiteler Mahallesi, Dumlupınar Bulvarı, Ankara, Turkey; Kyoto University, JAPAN

## Abstract

In this study, we focus on the possible roles of second-order syntactic recursion and working memory in terms of simple and complex span tasks in the development of second-order false belief reasoning. We tested 89 Turkish children in two age groups, one younger (4;6–6;5 years) and one older (6;7–8;10 years). Although second-order syntactic recursion is significantly correlated with the second-order false belief task, results of ordinal logistic regressions revealed that the main predictor of second-order false belief reasoning is complex working memory span. Unlike simple working memory and second-order syntactic recursion tasks, the complex working memory task required processing information serially with additional reasoning demands that require complex working memory strategies. Based on our results, we propose that children’s second-order theory of mind develops when they have efficient reasoning rules to process embedded beliefs serially, thus overcoming a possible serial processing bottleneck.

## Introduction

Theory of mind (ToM) is the ability to understand that people have mental states, such as desires, beliefs, knowledge and intentions, and to realize that mental states of others might be different from one’s own [1]. Zero-order ToM reasoning concerns our real-life environment. For instance, if David thinks: “There is a newspaper on the table”, he is applying zero-order reasoning. However, in daily life we are not just thinking about world facts. For example, David might think: “Jessica *knows* that there is a newspaper on the table”. In this situation David engages in first-order ToM by making a first-order knowledge attribution to Jessica. In addition to first-order ToM, there are higher orders of ToM, such as David thinking, “Jack *believes* that Jessica *knows* that there is a newspaper on the table”. This time, David is applying second-order recursion in the thought domain by attributing a first-order mental state to Jack.

First-order theory of mind has been found to be required for a number of simple social skills and competences. For example, children only start to be able to choose between informative ploys and deceptive ploys (such as removing tracks or adding false tracks) while hiding a toy when they are around the age of 4; their appropriate choices then correspond to first-order mental state attributions such as “now she will *not know* where the toy really is, but she will *think* it is under the cup where the tracks go” [2].

At the next step of development, second-order theory of mind has been found to be required for more advanced aspects of children’s everyday social competence, such as idiom understanding [3], which corresponds to second-order attributions like a hearer’s reasoning “Peter is not really skating on thin ice, so the speaker *wants* me to *think* of a different meaning”. As another concrete example, to successfully maintain a strategic lie, a lying child has to reason about what the listener *knows* about what the liar *knows*, requiring second-order theory of mind [4]. Similarly, second-order theory of mind is a prerequisite for more complex moral judgments such as “the father *knows* that his daughter *thinks* that he will go to the pool, so he should really go there” [5]. Finally, second-order theory of mind has been shown to be required for irony understanding (“although Oliver says ‘You sure are a great scorer’, Oliver doesn’t really *want* Robert to *believe* that he is a great scorer”) [6].

Dennett [7] argued that to have a theory of mind, a person has to be able to correctly attribute a false belief to someone else. Since then, verbal false belief tasks have become one of the most commonly applied tasks for testing theory of mind [8]. The goal of the first-order false belief task is to examine whether children can attribute a false belief to another person in a given story where the child knows the reality while the other person has a false belief about it. Similarly, the second-order false belief task examines whether children can correctly attribute to a person a false belief that that person has about another person’s belief. While first-order false belief understanding develops around the age of four [9], second-order false belief understanding develops between the ages of five and seven [10,11]. The goal of this study is to investigate four- to- eight-year-old children’s development of second-order false belief reasoning.

One striking and much debated finding is that there is a delay between first- and second-order false belief reasoning in middle childhood. Why do children need some more years to pass second-order false belief tasks once they are able to pass first-order false belief tasks? The answer to this question is not entirely clear yet. Following the first-order ToM literature, two possible explanations have been proposed (see e.g., [12], p. 751). The first explanation is related to a *conceptual change*: Children need to realize that mental states such as beliefs can have other beliefs and not just events in the world as their content (e.g., “John *thinks* that David *believes* that…”). The second explanation is related to the *complexity* of second-order ToM stories, in terms of the number of beliefs and their recursive organization. According to this explanation, it is the higher complexity of second-order ToM reasoning that adds further demands on working memory, as does the linguistic complexity of the stories and the questions, in comparison to first-order ToM tasks. Although we surmise that there might be a conceptual change of understanding that beliefs can have other beliefs as their content, in the current study we focus on the complexity explanation and aim to tease apart its components, namely executive functions and language.

For this purpose, for the first time in the literature, we focused on the role of working memory, together with the role of recursion in the language and thought domain on the same complexity and the same level of recursion, namely second-order.

In the following subsections of this Introduction, ‘Working Memory and Theory of Mind’ and ‘Syntactic Recursion and Theory of Mind’, we present relevant previous studies and provide theoretical explanations for the relations between working memory, language and second-order ToM.

### Working Memory and Theory of Mind

A number of other studies have shown that the development of executive function, more specifically working memory, influences the development of first-order ToM (e.g., [13–15]; but see also [16] for evidence that simple working memory is not sufficient without inhibition). On the other hand, to the best to our knowledge and according to Miller’s [12,17] extensive review, there are only two studies that focused on the role of working memory and second-order false belief reasoning in typically developing children and those studies yielded contradictory results.

The first one is Perner et al.’s [18] study with typically developing children and children at risk of ADHD. As a part of their study, to test executive function, they used forward and backward digit span. They found a significant positive relationship between the simple working memory task, i.e., the forward digit span, and the second-order false belief task. However, more recently, Hasselhorn, Mahler, and Grube [19] also tested children around the age of six with a second-order false belief task and with a simple working memory task (a digit span task) and a non-word repetition task together with verbal ability tasks. Their results showed that the significant correlation between the simple working memory span score and children’s second-order false belief score was no longer reliable when vocabulary knowledge and age were controlled for (*r*(56) = .13, *ns*).

What could be the role of working memory in the development of second-order false belief reasoning? It has been shown that working memory acts as a bottleneck [20], meaning that people can only hold one chunk of information in working memory at a time. Given this restriction, we invoke the *serial processing bottleneck* hypothesis [21].

Evidence for a working memory bottleneck in ToM reasoning comes from dual-task paradigms. The general idea of the dual-task paradigm is to find two different tasks and present them simultaneously, in order to compare performance with the two single tasks in which the participants have already performed well. If the performance of the first task decreases when it is presented concurrently with the second task, it can be inferred that the two tasks both require the same cognitive resource [22,23] and that that common resource acts as a bottleneck [20,24]. McKinnon and Moscovitch [25] showed that young adults performed significantly worse on second-order ToM reasoning than on first-order ToM reasoning in a dual-task condition with a demanding secondary executive function task (i.e., the 2-back task). Participants in the single-task condition who only did second-order ToM reasoning did not show any loss in performance. This result was replicated in another study with young adults using a more advanced and naturalistic ToM task, namely the “Reading the Mind in the Eyes” task [26].

The *serial processing bottleneck* hypothesis can be seen as a procedural explanation of the cognitive process of serializing the hierarchical content of thought into proper chunks along with their relations such that they fit easily through the processing bottleneck. Working memory constrains this process in terms of capacity and efficiency. The order of the processed chunks also reflects the reasoning steps children have to go through in order to solve a second-order false belief task. The *serial processing bottleneck* hypothesis is tantamount to a computational account under which second-order false belief can be conceived as a social-cognitive reasoning task employing a proper procedure–serialization–and a critical amount of mental resources–working memory–in order to cope with its nested structure.

Because working memory acts as a bottleneck, we propose that children who cannot pass second-order false belief tasks might have a lack of efficiency in reasoning when they have to serially process embedded beliefs. More specifically, when children try to answer second-order false belief questions, e.g., “Where does Mary think that John will look for the chocolate?” but have no efficient reasoning rule such as “if Mary didn’t see that John saw her hiding the chocolate, then she thinks that John thinks that the chocolate is still where he put it before, which is in the drawer”, more reasoning steps are needed to attribute a second-order false belief to Mary. A possible sequence of reasoning steps from the child’s perspective might be as follows: i) “John knows that the chocolate is in the toy box”, ii) “Mary does not know this”, iii) “Mary knows that John put the chocolate into the drawer before”, iv) “Mary thinks that John will look for the chocolate in the drawer”. In line with de Villiers et al. [27], each reasoning step is in the form of a single-embedded sentence at the surface. In addition to de Villiers et al.’s study, the *serial processing bottleneck* hypothesis allows us to propose a possible explanation for their failures on children’s development of processing these rules to answer the second-order false belief question in terms of working memory.

If children do not have an efficient reasoning rule, they need to go through each of the reasoning steps i-iv, which occupies working memory temporarily. In order to proceed in reasoning, due to the working memory bottleneck, at each step, the information in working memory needs to be sent to long-term memory to be retrieved later, if necessary. Retrieving information from long-term memory also takes time and increases the odds of forgetting and of retrieving wrong information [28]. Therefore, having more inefficient rules instead of one efficient rule means that the process is more prone to errors and takes more time [29,30]. This view is consistent with research showing that children perform better in language comprehension tasks and cognitive tasks when they are given more time [31–34]. Once children have enough experience in applying these reasoning steps sequentially, they are combined to one efficient rule, repeated here for convenience: “if Mary didn’t see that John saw her hiding the chocolate, then she thinks that John thinks that the chocolate is still where he put it before, which is in the drawer” (see [35] for the details of a mechanism that combines rules).

In order to investigate the *serial processing bottleneck* hypothesis, we looked at the relationships between a complex working memory task, a simple working memory span task, and second-order false belief reasoning. We did not use a dual-task paradigm because children have to be good at second-order false belief reasoning already in order to use a dual-task paradigm and our main focus is on children who are still developing second-order false belief reasoning. Although both simple and complex working memory tasks are related to working memory in a broader sense, they require different strategies. Unlike our simple working memory task that only requires building a representation of a list of words to be remembered, our complex working memory task requires processing information serially as well as cognitive control [36,37]. Therefore, we reason that children who have complex working memory strategies that can overcome the working memory bottleneck will be more successful in applying these in second-order false belief reasoning as well.

As a simple working memory span task, we used a word span task (WST) and as a complex working memory span task, we used a listening span task (LST). Considering the language-based nature of the listening span task, we used the word span task instead of the digit span task in order to keep the modality the same between the simple and complex span tasks. However, our results still can be compared with Perner et al.’s and Hasselhorn et al.’s study, because it has been shown that word span and digit span are closely related, *r* = .65, p < .001 [38] and have been grouped together with other span tasks which test the same component of working memory [39].

We follow Carlson’s [40] terminology, and refer to our simple word span task as a measure of pure working memory. Note that simple word span tasks have been referred to as short-term memory tasks as well [41]. The details of the tasks are explained in the Methods section.

### Syntactic Recursion and Theory of Mind

Similar to the studies showing that language development contributes to the development of first-order ToM [27,42–47] (but see [48] for evidence of false belief understanding in preverbal infants), a number of studies have shown that language is important in children’s development of second-order ToM.

For example, Hollebrandse, van Hout, and Hendriks [49] compared six- to nine-year-olds’ performance on a verbal and a low-verbal version of a second-order false belief task in order to investigate whether language in general helps children to pass second-order false belief tasks. They found that children’s scores were lower in the low-verbal version of the second-order false belief task compared to the verbal version and concluded that language might support explicit reasoning about higher-order beliefs by facilitating tracking different beliefs. Similar to the findings of Hollebrandse et al. [50], Kuijper [51] found that low-verbal second-order false belief tasks are also harder than high-verbal ones for children between six and twelve years of age who have been diagnosed with autism spectrum disorder or attention-deficit/hyperactivity disorder.

Lockl and Schneider [52] found that, at the age of five, children’s general language abilities (i.e., a combined score of sentence comprehension, morphological rule abilities and sentence memory) were strongly correlated with their second-order false belief reasoning. However, they stated that their data were not well suited to separate out the effects of syntactic and semantic abilities (p. 163). These studies indicate that explicit mental state language may support the development of second-order false belief reasoning. Yet, it is still not clear which aspect of language it is that helps.

Therefore, in this study, we investigate the possible relationship between syntactic recursion in the language domain and recursion in the thought domain on the same level of recursion, namely second-order.

The syntactic component of language is found to be related to first-order ToM in terms of its hierarchical embedding structure [27,43,53–55]. Usually, first-order complement clauses, as shown below in Example (1) (adapted from [44]), have been used in the literature to investigate this relationship. Complement clauses such as “*that p*” may be used to express propositional attitudes (or opinions) towards some state p in the world [27]. They may be preceded by mental state verbs as in “Mary knows *that* p” or in “Mary believes *that* p” or by communication verbs as in “Mary said *that* p”. Complement clauses can be used recursively, as shown below in Example (2). Moreover, complement clauses allow people to represent states that contrast with reality or with other people’s mental states in terms of truth-value. Thus, while in Example (2), “Mary said that there was a flea in her cereal” might be false, the whole sentence “John said that Mary said that there was a flea in her cereal” might be true.

1. First-order complement clause: “Mary said *that there was a spider in her cereal*. But it was just a raisin”.
2. Second-order complement clause: “John said *that Mary said that there was a flea in her cereal*. But in fact, she said *that there was a spider in her cereal*”.

Recently, de Villiers et al. [27] argued that experience with truth-value contrasts in contexts with full tensed complement clauses such as Example (1) opens the door for children to pass first-order ToM tasks and to recognize syntactic recursion. Subsequently, understanding sentence recursion allows children to pass recursive ToM tasks. More specifically, they suggest that recursive complements in contexts in which the truth-values vary, such as in Example (2), are necessary for recursive false belief reasoning. However, they conclude that their predictions need to be explored further.

Like complement clauses, *relative clauses* can be used recursively; that is, besides first-order relative clauses, there are second-order relative clauses as well as even higher-order relative clauses. At each level of recursion they may refer to a different subject or object. However, unlike complement clauses, relative clauses do not involve propositional attitude verbs such as “knowing *that*” or “believing *that*” or communication verbs such as “saying *that*” and they do not involve truth-value contrasts. Table 1 shows examples of the progression of orders of recursion for ToM attributions and relative clauses. Unlike de Villiers et al. [27], who focused on complement clauses, we used relative clauses, which allows us to specifically focus on the structural parallelism between second-order recursion in the language domain and in the thought domain by excluding the role of truth-value contrasts.

**Table 1:** Examples of the progression of orders of recursion for theory of mind attributions and relative clauses.

Levels of recursion	Theory of mind	Relative clauses
Zero-order	The sheep is pushing a monkey.	The sheep is pushing a monkey.
First-order	You think that [the sheep is pushing a monkey].	You show me [the sheep that is pushing a monkey].
Second-order	You think that [the monkey thinks that [the sheep is pushing a monkey]].	You show me [the monkey that is pushing [a sheep that is pushing a monkey]].

In the first-order domain, Hale and Tager-Flusberg [56] demonstrated that preschoolers who were trained on first-order complement clauses improved their first-order ToM skills significantly while those trained on first-order relative clauses did not. On the other hand, Smith, Apperly, and White [57] found a positive correlation between first-order relative clauses and first-order false belief tasks in children between the ages of 3 and 4. They concluded that first-order false belief reasoning might not be related to the specific structure of the complement clauses but to the broader category of embedded structures. Another developmental study with Turkish first-order relative clauses supports this positive relationship of first-order relative clauses and first-order ToM [58].

Although the role of first-order relative clauses in first-order false belief reasoning has not been resolved yet, it is worthwhile to investigate the relationship between second-order relative clauses and second-order false belief reasoning. This is because relative clauses share only the *syntactic* feature of embedded (meta-) representation with second-order false belief reasoning, in contrast to complement clauses, which additionally share semantic features with false belief, namely the fact that the main clause and the embedded complement clause have independent truth-values [56,57,59]. Since we are mainly interested in the structural and representational parallelism between recursion in the language and the thought domain, we chose a second-order relative clause comprehension task (REL_2) as a linguistic predictor of a second-order false belief task, which importantly concerns the same level of recursion. To the best of our knowledge, this is the first time that second-order relative clauses have been studied in relation to second-order false belief reasoning.

### Predictions

1. Based on the *serial processing bottleneck*, we predict that the relationship between the complex working memory task and the second-order false belief task will be stronger than the relationship between the simple working memory task and the second-order false belief task.
2. Because the second-order false belief task and the second-order relative clause task share the same level of recursion, we expect a significant correlation between the two tasks.

Therefore, we expect both the complex working memory task and the second-order relative clause task to figure importantly in the development of second-order false belief reasoning.

## Method

### Participants

Initially, a sample of 103 children between the ages three and eight was recruited from local kindergartens and primary schools in predominantly middle- and upper middle-class areas of Ankara, Turkey. Our study has been approved by Middle East Technical University (METU) Research Centre for Applied Ethics. A written parent approval form was obtained for every child that participated in the study. All children were monolingual Turkish native speakers. Three children left the study before it was completed. We excluded the youngest 11 children (range: 3;8–4;5) because they had very low scores in all tasks, indicating that the tasks were too hard for them in general. Thus, the results of 89 children were analyzed (37 female, *M*_*age*_ = 6;7 years, *SE* = 0.13, range: 4;5–8;10). Gender did not show any effect; therefore, the analyses were collapsed over gender. Considering the previous literature which indicates that second-order false belief reasoning starts to manifest itself between the ages five and seven [10,11] and that Turkish children’s development of first-order and second-order false belief reasoning shows a similar pattern with children in Western countries [58,60,61], we divided participants into the following two age groups: children younger than 6;6 were assigned to a *younger* age group and children older than 6;6 to an *older* age group:

1. *Younger (4–6 years) n* = 41; range = 4;6–6;5; *M*_*age*_ = 5;6; *SE* = 0.10; 17 female,
2. *Older (6–8 years) n* = 48; range = 6;7–8;10; *M*_*age*_ = 7;6; *SE* = 0.11; 20 female.

### Design

A cross-sectional study design was used with age as a quasi-independent between-subjects variable. The same person tested all of the children in a quiet empty classroom at their school. For each child, all of the tests were completed in one session, which varied from 25 to 35 minutes.

All children participated in the following four tests in the following order: Word span task, second-order false belief tasks, second-order relative clause task, and listening span task. We wanted to keep some temporal distance between the two working memory tasks to prevent any interference effects. For this reason, we used one working memory task at the beginning and the other at the end of the session. Moreover, because we used children’s second-order false belief task scores as dependent variables, we used this task as a second task in our study, in order to prevent a possible fatigue effect. As Carlson and Moses [62] argued, using a fixed order of tasks is standard practice in individual differences research and especially for interpreting correlations between tasks it is important that the task order remains the same for all participants.

Note that we also tested children with a pragmatic understanding task that we constructed. This task was presented after the second-order false belief tasks. In this task, children had to choose correct morphological case markers on an object–definite or indefinite–depending on whether the protagonists in a story had encountered the object before or not. However, this task did not correlate with any other task that we used in the experiment. For this reason, we do not present this task and its results here.

### Materials and Procedure

In this section, we present the materials and the procedures in the following order: second-order false belief task, second-order relative clause task, word span task, and listening span task. All of the stimuli can be found in S1 Materials.

#### Second-order false belief task (FBT_2)

This task consists of two different second-order false belief stories, namely the “Birthday Puppy Story” and the “Chocolate Bar Story”. Both stories were adapted from English to Turkish from Flobbe and colleagues’ [63] study with the authors’ permission. These stories were told to the subjects while presenting Flobbe and colleagues’ drawings. Second-order embedding structures such as “Ayla thinks that Murat thinks that the chocolate is in the drawer” were not explicitly used in the stories. The order of stories was balanced.

While hearing a story, children were first asked a reality control question (“Where is the chocolate now?”), and a first-order ignorance question (“Does Murat know that Ayla has hidden the chocolate in the toy chest?”), as well as a linguistic control question (“Does Ayla know that Murat saw her hide the chocolate?”). The experimenter repeated the essential parts of the story and the control questions if a child gave a wrong answer for the control questions to make sure that children did not have any problems with remembering the stories and with the syntactic structure of double-embedded clauses. The upper limit for repeating the story and the control questions was three times (see [6,64] as an example for repeating the control questions). All of the children gave correct answers to the control questions within that limit.

After the control questions, the children were asked (only once) a second-order false belief question: “Where does Ayla think that Murat will look for the chocolate?” and then (only once) a justification question: “Why does Ayla think that?”. In order to investigate the effects of our syntactic recursion and working memory tasks at different stages of second-order ToM development, we analyzed children’s judgments of the second-order false belief questions and justifications for their judgments separately (see [65], for an example of reporting judgment and justification answers separately). The rationale for analyzing both judgment and justification answers derives from a computational study showing that the operational demands of providing a justification are higher than the demands of making a judgment [66]. Therefore, it might be possible that the syntactic recursion and working memory have different effects on children’s judgments and justifications in second-order false belief tasks. A judgment score of 1 was given for a correct answer to a second-order false belief question, and a score of 0 was given for a wrong answer and for the answer “I don’t know”. Because we used two different stories, judgment scores could range from 0–2.

Children’s justifications for the second-order false belief task were coded based on the methods described by Perner and Wimmer [10] and Sullivan et al. [11]. The categories were divided into two groups: correct and incorrect. If a child’s justification answer included the correct information that one character does or does not know about the other character’s history of exposure to relevant information, it was coded as correct. Otherwise, the justification was coded as incorrect (0 points). Correct justifications were divided into the following five mutually exclusive groups:

1. *Explicit second-order reasoning*: The child embeds one character’s epistemic state in the other character’s mental state, for example, “Because she believes that Murat doesn’t know that the chocolate is in the box” (“Çünkü Murat’ın çikolatanın kutunun içinde olduğunu bilmediğini zannediyor”).
2. *Implicit second-order reasoning*: Relevant information is embedded in one character’s epistemic state, for example, “Because she doesn’t know that Murat saw it” (“Çünkü Murat’ın gördüğünü bilmiyor”). Similar to Sullivan et al.’s [11] study, we consider this statement to be second-order because of the role it plays in justifying a correct response.
3. *Perceptive information*: Relevant information is embedded in one character’s perception, for example, “Because she didn’t see that Murat was looking through the window” (“Çünkü Murat’ın pencereden baktığını görmedi”).
4. *Communicative information*: Information is mentioned that was communicated to the secondary character, for example, “Because she said she bought a ball” (“Çünkü top aldığını söylediği için”).
5. *Location information*: The original location of the object is mentioned, for example, “Because Murat put it into the drawer before” (“Çünkü Murat çekmeceye koymuştu”).

As can be seen from the above-mentioned groups of justification answers, the sophistication of the answers differs. While the answers in the explicit and implicit second-order reasoning groups (a and b) include mental state words, the other three groups (c, d and e) do not include any mental state word. For these reasons, we gave 2 points for the answers in the explicit and implicit second-order reasoning categories and we gave 1 point for the answers in the other categories (see [6] for an example of a similar scoring procedure as ours, distinguishing different types of justifications based on the complexity of the answers). Because children were tested with two stories, the score range for their justifications was 0–4 in total.

Note that originally, we constructed three different versions of each second-order false belief story, in order to investigate the effect of three morphological evidential markers on the understanding of children’s second-order false belief reasoning: Neutral (present tense),–DI (past tense indicating direct perceptual evidence),–mIş (past tense indicating hearsay or inference). Only one of these versions was presented to each subject (see S1 Materials for details). Evidential markers encode the source of information and may therefore allow speakers and learners of evidential languages such as Turkish to take a positional perspective on a given propositional content–similar to propositional attitudes in false belief tasks [67]. However, because we did not find any significant difference between the three evidential conditions, we collapsed the data over them.

#### Second-order relative clause task (REL_2)

**Fig 1:**
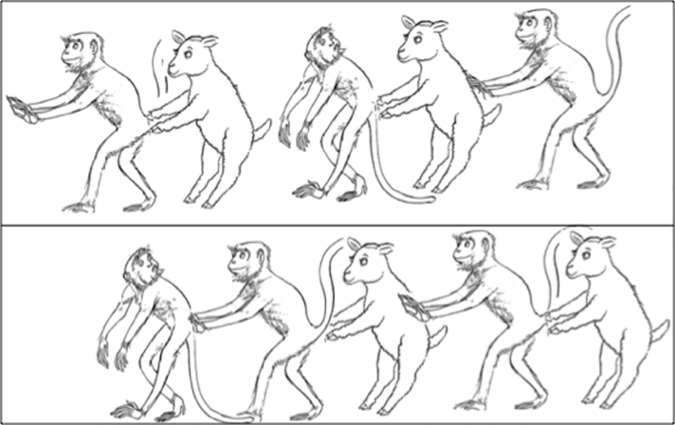
Picture used in the second-order relative clause task (REL_2). “In which picture is there a sheep that is pushing a monkey that is pushing a sheep?” Adapted from Özge et al. [68] under a CC BY license, with permission from the authors.

This task concerns the comprehension of relative clauses in Turkish and was adapted from Özge, Marinis, and Zeyrek’s [68] first-order relative clause task with the authors’ permission. The questions and the drawings were modified to second-order relative clauses. Fig 1 demonstrates the drawings for one of the questions in this task. First, introductory pictures were shown to the participants in order to familiarize them with the animals in the actions by telling the name of the animals and the actions (e.g., “This is a pushing sheep, this is a looking monkey and this is a pushing monkey”). After that, the pictures representing the questions were shown one by one. The first and second rows of the picture were pointed out in order to make clear that there were two separate lines of pictures by saying: “This is the first picture and this is the second picture”. In the practice session, the experimenter explained that the participants were required to point out the row with the animals corresponding to their answer. If they could not answer correctly in the practice session, the experimenter pointed out the correct animals and described their actions. However, no feedback was provided during the experimental session. The sentences were repeated up to 4 times. The critical positions for finding the correct answers were equally distributed across the drawings (3 times in the first row and 3 times in the second row) and between right (2 times), left (2 times), and central position (2 times). One practice item and 6 experimental items were used. A child’s total score for experimental items was minimally 0 and maximally 6.

There are other types of relative clauses, e.g., “You show me the sheep that a monkey is pushing that a sheep is pushing”. Because our aim is to examine the relationship between syntactic recursion and second-order false belief reasoning and not children’s different abilities in different types of relative clauses, we used relative clauses of the form “In which picture is there a sheep that is pushing a monkey that is pushing a sheep?”, which are straightforward to understand [68]. Children are exposed to recursion in relative clauses from an early age, for example in well-known nursery rhymes such as “This is the house that Jack built”, also cited in de Villiers and de Villiers [44]: “This is the maiden all forlorn, that milked the cow with the crumpled horn, that tossed the dog that worried the cat, that chased the rat that ate the cheese, that lay in the house that Jack built.”

Note that one might argue that children tend to interpret indirect recursion as conjunction reading. Thus, they might interpret “In which picture is there a sheep that is pushing a monkey that is pushing a sheep?” as “In which picture is there is a sheep that is pushing a monkey *and* a sheep?”. However, as you can see in Fig 1 and in S1 Materials, none of the pictures allow for such a conjunctive reading. Because all of our subjects pointed out three adjacent animals in one of the pictures throughout the task, we can say that this argument is ruled out.

#### Word span task (WST)

Children’s simple working memory span was tested using a Turkish version of the word span task (WST) [69]. Monosyllabic Turkish words such as “saç”, “tuz” and “yurt” (hair, salt and country) were selected, considering their frequency in daily usage and ease of pronunciation. There were seven sets that corresponded to ascending levels of difficulty. Each level *k* contained three subsets of *k*+1 words each. At the first level, there were three subsets of two words each, and at the seventh (last) level, there were three subsets of eight words each. An example of the first level is: i) köşk–muz (manor—banana); ii) pil– üst (battery-upper); iii) buz–dört (ice–four), and an example of the seventh (last) level is: i) tam–bak–uç –göz–hal–boş –ek–yurt; ii) üç –kas–al–mülk–bir–tut–dil–kum; iii) bul–pek–on–fal–var–el–ses–genç. The words from these levels were read to the participants, starting from the first subset at the first level. After reading one subset (e.g., köşk–muz), the participant repeated the words in that order. If the participant could not correctly reproduce two out of three subsets at level *k*+1, the task was terminated and the level *k* was the score of the participant. Thus, in the analysis, a child’s word span range may vary between 0 and 7.

#### Listening span task (LST)

Children’s complex working memory span was tested using a Turkish version of the listening span task (LST) [69]. The task consisted of sets of sentences read out to the participants one by one. There was a total of five collections, each of which consisted of six sets of sentences. The first collection contained six sets of two sentences each, the second collection contained six sets of three sentences each, and so forth, until the fifth collection, which contained six sets of six sentences each. An example of a 2-sentence set of LST is as follows: i) Muzlar bisiklete biner (“Bananas ride bicycles”); ii) Elimiz beş parmaklıdır (“Our hands have five fingers”). The participants were expected to first judge the truthfulness of each sentence by saying “Yes” or “No”. Secondly, they had to recall the last word of all the sentences of a set told to them so far, in reverse order. After they gave an answer to the first sentence, the next sentence was told to them. For example, for the 2-sentence set, if the first sentence was “Muzlar bisiklete biner” (“Bananas ride bicycles”), the participants were required to say “Hayır; biner” (“No; bicycles”). After that, if the second sentence was “Elimiz beş parmaklıdır” (“Our hands have five fingers”), they were required to say “Evet; parmaklıdır, biner” (“Yes; fingers, bicycles”). If the participant made at most one mistake in a sentence collection, the subsequent sentence collection, which comprised one more sentence per set, was told to the participant. The score of the participants equaled the number of sentence collections in which they did not make more than one mistake. Thus, participants’ scores could range from 0–6.

## Results

Our main goal was to investigate the role of language and working memory in the development of second-order ToM. In more detail, for the role of working memory, we aimed to test the *serial processing bottleneck* hypothesis by using both a complex and a simple working memory span task. Based on the *serial processing bottleneck* hypothesis, we predicted that the relationship between the complex working memory task and the second-order false belief task will be more salient than the relationship between the simple working memory task and the second-order false belief task. Because second-order relative clauses have the same level of recursion, we hypothesized that a child’s score on the second-order relative clause task would be a predictor of his or her second-order false belief scores.

The second-order false belief task (FBT_2) judgment scores (*W* = 0.77, *p* < .001) and the justification scores (*W* = 0.782, *p* < .001) were non-normally distributed. Because the data violated the normality assumption of ANOVA, a cumulative odds ordinal logistic regression with proportional odds was run [70]. The proportional odds and multicollinearity assumptions were satisfied. We first report the results for the development of tasks individually. We then report the bivariate and partial correlations among the tasks. Finally, we predict the FBT_2 judgment and FBT_2 justification scores by using ordinal logistic regression. Note that all the effect sizes (*B*) in the ordinal logistic regression are in terms of log odds.

### Testing the Differences between Younger (4–6) and Older (6–8) Age Groups

Table 2 presents the means together with the standard deviations of all the variables for each age group. Note that children between the ages 4;6 and 6;6 were assigned to the *younger* age group and between the ages 6;7 and 8;10 were assigned to the *older* age group:

**Table 2:** Descriptive statistics of the four tasks administered to each age group.

	Younger (4–6 years)	Older (6–8 years)
Tasks	Mean (SD)	Mean (SD)
False belief judgment (Range 0–2)	1.04 (0.82)	1.54 (0.58)
False belief justification (Range 0–4)	1.10 (1.30)	1.29 (1.27)
Relative clause task (REL_2) (Range 0–6)	1.15 (1.49)	1.92 (1.78)
Word span task (WST) (Range 0–8)	4.05 (0.80)	4.81 (0.87)
Listening span task (LST) (Range 0–6)	0.34 (0.69)	1.13 (0.98)

The analyses showed that while children in the older (6–8 years) group outperformed children in the younger (4–6 years) group in the FBT_2 judgment scores (*B =* 1.29, *SE =* 0.42, *p* = .002), there was no significant difference in the FBT_2 justification scores between the younger and older age groups (*B =* 0.35, *SE =* 0.39, *p* = .38). Moreover, children in the older group outperformed children in the younger group for the REL_2 task, (*B =* 0.88, *SE =* 0.39, *p =* .03), for the WST task, (*B =* 1.69, *SE =* 0.45, *p <* .001), and for the LST task, (*B =* 1.87, *SE =* 0.47, *p* < .001).

Table 3 shows the number of participants and percentages (in parentheses) for each second-order false belief (FBT_2) judgment score (0–2) and justification score (0–4). Consistent with the literature [17], judgment scores for the second-order false belief question were a bit higher for the ‘Birthday Puppy’ story than for the ‘Chocolate Bar’ story for both the younger age group (*M*_*score*.*chocolate bar*_ = 0.46, *SD* = 0.50; *M*_*score*.*birthday puppy*_ = 0.56, *SD* = 0.50) and the older age group (*M*_*score*.*chocolate bar*_ = 0.69, *SD* = 0.47; *M*_*score*.*birthday puppy*_ = 0.85, *SD* = 0.36).

**Table 3:** Number of participants and percentage (in parentheses) of each second-order false belief (FBT_2) score.

	FBT_2 judgment score			FBT_2 justification score				
Age Group	0	1	2	0	1	2	3	4
Younger (4–6)	13 (32%)	14 (34%)	14 (34%)	20 (49%)	5 (12%)	12 (29%)	0 (0%)	4 (10%)
Older (6–8)	2 (4%)	18 (38%)	28 (58%)	18 (37%)	10 (21%)	11 (23%)	6 (13%)	3 (6%)

The detailed results about the frequency and percentage of each type of justification answer are shown in Table 4. As can be seen from Table 4, children’s correct justification answers mostly involved implicit second-order answers (e.g., “Because she doesn’t know that Murat saw it”) for both age groups. Moreover, while there were two children in the older age group who gave explicit second-order justification answers (e.g., “Because she believes that Murat doesn’t know that the chocolate is in the box”), none of the children in the younger age group gave any explicit second-order answers. However, children’s justification answers in the older age group (6–8) are clearly not at the ceiling and probably continue to develop after the age of 8.

**Table 4:** Frequency (Freq.) and percentage (%) of each type of justification answers.

Story type	Justification type	Younger (4–6 years)		Older (6–8 years)	
		Frequency	%	Frequency	%
Chocolate Bar story					
	Explicit second-order	0	0	1	2
	Implicit second-order	13	32	10	21
	Perceptive information	1	2	9	19
	Communicative information	0	0	0	0
	Location information	2	5	6	12
	Wrong answers	25	61	22	46
	Total	41	100	48	100
Birthday Puppy story					
	Explicit second-order	0	0	1	2
	Implicit second-order	7	17	10	21
	Perceptive information	0	0	0	0
	Communicative information	2	5	3	6
	Location information	0	0	0	0
	Wrong answers	32	78	34	71
	Total	41	100	48	100

### Bivariate and Partial Correlations

To inspect the interrelationships among the four tasks, we conducted bivariate and partial correlations. Table 5 shows the bivariate correlation coefficients (Spearman’s *r*_*s*_) and Table 6 shows the partial correlation coefficients (Spearman’s *r*_*s*_) for the younger (4–6 years) and older (6–8 years) groups. We used age (in months) as a control variable.

**Table 5:** Bivariate correlation coefficients (Spearman’s *r*_*s*_) for the younger (4–6 years) and older (6–8 years) age groups.

		Younger group (4–6 years)					Older group (6–8 years)				
		1^a^	2	3	4	5	1	2	3	4	5
FBT_2 Judgment (range: 0–2)	1. Age (in months)	-	-	-	-	-	-	-	-	-	-
	2. Judgment	.27	-	-	-	-	.13	-	-	-	-
	3. Relative clause task (REL_2)	.14	.33*	-	-	-	.001	.09	-	-	-
	4. Word span task (WST)	.15	.15	.32*	-	-	.15	.13	.42**	-	-
	5. Listening span task (LST)	.10	.51***	.66***	-.05	-	.15	.17	.41**	.38**	-
FBT_2 Justification (range: 0–4)	1. Age (in months)	-	-	-	-	-	-	-	-	-	-
	2. Justification	.14	-	-	-	-	.21	-	-	-	-
	3. Relative clause task (REL_2)	.14	.48**	—	-	-	.001	.22	-	-	-
	4. Word span task (WST)	.15	.29	.32*	-	-	.15	.15	.42**	-	-
	5. Listening span task (LST)	.10	.59***	.66***	-.05	-	.21	.37**	.41**	.38**	-

Note.

* *p* < .05

** p < .01

*** *p* < .001.

^a ^The numbers in this row are used as abbreviations for the age and the tasks that were enumerated in the second column of this table.

**Table 6:** Partial correlation coefficients (Spearman’s *r*_*s*_) for the younger (4–6 years) and older (6–8 years) groups.

		Younger group (4–6 years)				Older group (6–8 years)			
		1^a^	2	3	4	1	2	3	4
FBT_2 Judgment	1. Age (in months)	-	-	-	-	-	-	-	-
	2. Relative clause task (REL_2)	.31*^b^	-	.30*	- .001	.09	-	.03	.02
	3. Word span task (WST)	.12	.05	-	.20	.12	.11	-	.08
	4. Listening span task (LST)	.50***	.41**	.52***	-	.15	.14	.13	-
FBT_2 Justification	1. Age (in months)	-	-	-	-	-	-	-	-
	2. Relative clause task (REL_2)	.47***	-	.43**	.16	.22	-	.08	-
	3. Word span task (WST)	.27	.16	-	.39**	.12	.06	-	.004
	4. Listening span task (LST)	.58***	.41**	.63***	-	.34*	.32*	.35*	-

Note.

* *p* < .05

** p < .01

*** *p* < .001.

^a ^The numbers in this row are used as abbreviations for the age and the tasks that were enumerated in the second column of this table.

^b^ The partial correlations show the correlation between a variable in a row and judgment/justification score when a variable in a column is controlled for. For example, (.31*) shows the partial correlation between REL_2 and judgment score when age (in months) is controlled for.

As can be seen from Table 5, for the younger group (4–6), there is a significant correlation between the FBT_2 judgment score and REL_2 (*r*_*s*_ = 0.33, *p =* .03), and a significant correlation between the FBT_2 judgment score and LST (*r*_*s*_ = .51, *p* = < .001). However, as shown in Table 6, correlations between the FBT_2 judgment score and REL_2 become insignificant when we control for LST (*r*_*s*_ = —.001, *p* = .99) but remain significant when we control for age (*r*_*s*_ = .31, *p =* .04) and for WST (*r*_*s*_ = .30, *p* = .04). On the contrary, the correlation between the FBT_2 judgment score and LST remains significant when we control for age (*r*_*s*_ = .50, *p* < .001), REL_2 (*r*_*s*_ = .41, *p* = .006) and WST (*r*_*s*_ = .52, *p* < .001). For the older group (6–8), none of the tasks show significant correlations with the FBT_2 judgment score. The lack of significant relationships between LST and older children’s FBT_2 judgment scores (range 0–2) is due to the fact that the older children already performed well in providing judgment answers, so there is a lack of enough variation in the data.

Similar to the FBT_2 judgments scores, for the younger age group, there is a significant correlation between the FBT_2 justification score and REL_2 (*r*_*s*_ = .48, *p =* .001), and a significant correlation between the FBT_2 justification score and LST (*r*_*s*_ = .59, *p <* .001). Moreover, there is a marginally significant correlation between the FBT_2 justification score and WST (*r*_*s*_ = .29, *p =* .07). As partial correlations reveal in Table 6, only the correlation between the FBT_2 justification score and LST remains significant when we control for age (*r*_*s*_ = .58, *p <* .001), REL_2 (*r*_*s*_ = .41, *p =* .006) and it even increases somewhat when we control for WST (*r*_*s*_ = .63, *p <* .001), previewing the results of the subsequent regression analyses.

For the FBT_2 justification scores in the older age group, there is only a significant correlation between the FBT_2 justification score and LST (*r*_*s*_ = 0.37, *p =* .009). As shown in Table 6, the correlation between the FBT_2 judgment score and LST remains significant when we control for age (*r*_*s*_ = .34, *p* = .01), REL_2 (*r*_*s*_ = .32, *p* = .02) and WST (*r*_*s*_ = .35, *p* < .01).

Moreover, for both FBT_2 judgment and justification scores, we found a significant high correlation between LST and REL_2 (*r*_*s*_ = .66, *p* < .001). These correlations are still significant when we control for age and the other tasks that we used in this study, indicating that LST and REL_2 share a considerable amount of variance.

### Predicting the Second-Order False Belief Task Score (FBT_2)

The results of the regression models that best predict second-order false belief judgment and justification scores for both the younger (4–6 years) and the older (6–8 years) age groups are presented in Table 7. We proceeded as follows with our model construction for the younger group: In model 1, we entered the control variable “age in months” in order to account for any more fine-grained age differences within the younger age group, as well as the second-order relative clause task (REL_2), and the complex working memory task (LST), because we had found significant bivariate correlations between the FBT_2 judgment score and these two tasks. Moreover, even though we did not find strong significant correlations between the simple working memory task (WST) and FBT_2 judgment and justification scores, we kept WST in our models because we had a specific prediction that LST is more related to second-order false belief reasoning than WST is, based on the *serial processing bottleneck* hypothesis. In Model 1, the effects of age in months (*B =* 0.05, *SE =* 0.04, *p =* .22), REL_2 (*B =* -0.51, *SE =* 0.36, *p =* .16), and WST (*B =* 0.68, *SE =* 0.46, *p =* .14) were insignificant; only the effect of LST was significant (*B =* 3.44, *SE =* 1.35, *p =* .01). As shown in Table 5 and Table 6, considering the strong correlations between REL_2 and LST, and based on the result of Model 1 that REL_2 is not significant, we constructed Model 2 by excluding REL_2 from Model 1. As can be seen from Table 7, the effect of LST on the FBT_2 judgment score is significant (*B =* 2.22, *SE =* 0.82, *p =* .007), and the effect of WST is insignificant (*B =* 0.39, *SE =* 0.40, *p =* .33). The model comparison of Model 1 (Akaike Information Criterion (*AIC*) = 82.60) and Model 2 (*AIC* = 82.77) was less than 2. Because a simpler model is preferred over a more complex one, we presented the results of Model 2 in Table 7.

**Table 7:** Predicting second-order false belief task judgment and justifications scores for both younger (4–6) and older (6–8) age groups.

	Younger group (4–6 years)					Older group (6–8 years)			
	Variable	*B*	*SE*	*t*	*p*	*B*	*SE*	*t*	*p*
FBT_2 Judgment	Age (in months)	0.06	0.04	1.48	.138	0.03	0.03	0.82	.41
	Word span task (WST)	0.39	0.40	0.97	0.33	0.08	0.36	0.21	.83
	Listening span task (LST)	2.16	0.82	2.67	.008	0.27	0.32	0.83	.41
FBT_2 Justification	Age (in months)	-0.005	0.04	-0.10	.92	0.03	0.03	1.02	.31
	Word span task (WST)	1.12	0.49	2.29	.02	-0.05	0.34	-0.16	.88
	Listening span task (LST)	1.91	0.57	3.35	< .001	0.79	0.33	2.42	.02

We followed the same procedure as explained above for the prediction of the younger age group’s FBT_2 justification scores. We entered the control variable “age in months”, REL_2, LST, and WST into Model 3. In Model 3, the effects of age (*B =* - 0.006, *SE =* 0.04, *p =* .89), and REL_2 (*B =* - 0.10, *SE =* 0.30, *p =* .75) were insignificant, and the effects of LST (*B =* 2.05, *SE =* 0.73, *p =* .005), and WST (*B =* 1.18, *SE =* 0.53, *p =* .03) were significant. Based on the results of Model 3, we constructed Model 4 by excluding REL_2 from Model 3. As can be seen from Table 7, the effects of LST (*B =* 1.91, *SE =* 0.57, *p <* .001) and WST (*B =* 1.12, *SE =* 0.49, *p =* .02) on the FBT_2 justification score are significant. Because Model 4 (*AIC* = 90.04) is a simpler model than Model 3 (*AIC* = 91.94), we presented the results of Model 4 in Table 7.

For the older group (6–8 years), we had found a significant correlation only between LST and the FBT_2 justification score, and for them, none of the tasks were significantly correlated with the FBT_2 judgment score due to the lack of variation. Although none of the tasks were significantly correlated with the FBT_2 judgment score, because the younger group’s (4–6 years) final model to predict the judgment score includes LST and WST, we constructed Model 5 to predict the older group’s FBT_2 judgment score by adding LST and WST. As shown in Table 7, in line with the lack of significant correlations, both LST and WST’s effects were insignificant in the model.

Similar to the previous procedures, in order to predict older children’s FBT_2 justification scores, we constructed Model 6 by entering the control variable “age in months”, WST, and LST. The effect of LST is significant when we control for age (*B =* 0.79, *SE =* 0.33, *p =* .02), and the effect of WST is insignificant (*B = -*0.05, *SE =* 0.34, *p =* .88).

These results suggest that the main predictor of second-order false belief reasoning is not syntactic recursion and word span task but complex working memory.

## Discussion

The main goal of this study was to investigate the role of syntactic recursion and working memory in the development of second-order false belief reasoning as well as to provide a procedural account for the role of working memory. In order to focus on the different stages of children’s development of second-order false belief reasoning, we have run separate analyses of children’s judgments for the second-order false belief question and justifications for their judgments. Our results showed that the main and strongest predictor of the development of the second-order false belief reasoning is the complex working memory span (LST).

Let us first discuss the results related to the simple and the complex working memory tasks. Considering the literature that we discussed in the Introduction, our finding that there is no significant correlation between our simple working memory task and the second-order false belief judgement scores for both younger (4–6 years) and older (6–8 years) age groups is in line with Hasselhorn et al.’s study [19] that found no significant correlation between the simple working memory task and the second-order false belief task when the effects of verbal ability tasks and age were controlled for. Furthermore, our findings are also consistent with the previous literature that shows that complex working memory tasks are better predictors of measures of general intelligence than simple working memory tasks [71,72]. However, as shown in Table 7, in addition to the highly significant effect of the complex working memory task, the simple working memory task explains significant variation in younger children’s (4–6) justification answers. This significant correlation of the simple working memory task disappears for older children and only the complex working memory task is able explain the variance in children’s justification answers. As we predicted, we found a significant correlation between our complex working memory task and the second-order false belief judgment and justification scores, even when we controlled for the simple working memory task, the second-order relative clause task and age in months–in the younger age group. Moreover, for the older age group, for the complex working memory task, we found that it only significantly predicts the second-order false belief justification score, not the second-order judgment score. The reason is that the judgment scores do not differ much among the older children, while their justifications still do. Thus, justifications seem to be a more sensitive variable for older children in the sense that they provide a finer distinction in their second-order reasoning abilities. While older children can give correct second-order false belief answers, their development still continues in terms of their justification abilities.

Now, let us discuss our results in terms of one of our main goals, namely, testing the *serial processing bottleneck* hypothesis [21]. Why is the complex working memory task more important than the simple working memory task in predicting children’s performance on second-order false belief reasoning, in terms of both judgment and justification scores? The *serial processing bottleneck* hypothesis predicts that the difficulty of passing a second-order false belief task is not just related to holding the different beliefs in mind but also to serially processing them. In order to test this prediction, we have used both a simple working memory task (WST) that requires just holding the information in mind and a complex working memory task (LST) that requires not only holding information in mind but also processing that information serially, as well as additional reasoning demands that require complex working memory strategies. We argue that these differences between the complex working memory and simple working memory tasks could be the reason why the simple working memory task cannot explain enough variation of children’s performance on second-order false belief reasoning. Two subtasks appear to be required for successful second-order false belief reasoning: (1) keeping in mind the two separate beliefs (e.g., of John and Mary) and (2) mapping their nested, recursive structure onto the appropriate sequential order: Mary’s belief that John believes that p, such that they can pass the *serial processing bottleneck* smoothly. A few young children and many older children overcome the *serial processing bottleneck* by means of their complex working memory strategies, which are necessary for both the complex working memory task and the second-order false belief reasoning.

It is important to discuss some additional challenges of the Turkish version of the complex working memory task that we used, namely the listening span task. First, Turkish is a verb-final language–hence, the final word of the sentence, which is the critical word to be reported in the listening span task, may be a verb. Verbs and nouns have different semantic and computational loads and may therefore not be memorized equally well in the listening span task. Second, because in Turkish, the present form of the verb takes the suffixes–er,–ar,–ir, –ür,–ur (depending on vowel harmony) for positive sentences while it takes the suffixes–maz,–mez for negative sentences, an additional challenge of the task for children in our study was to repeat the last word of the sentence when the sentence was false and they had to say “Hayır” (“No”). So, participants had to inhibit the negative form of the final verb, e.g., for the sentence “Muzlar bisiklete biner” (“Bananas ride bicycles”) they should not say “binmez” (“they don’t ride”) but “biner” (“they ride”). This additional load in inhibition, due to the way the Turkish morphological system works, may cause the listening span task results to be somewhat different for Turkish children than for English-speaking children. Moreover, similar to what is argued in Carlson, Moses, and Breton [16] and Moses, Carlson, and Sabbagh [73] for the relation between first-order ToM and executive function, the additional inhibition demands in the Turkish version of the listening span task might be one of the reasons for its predictive power of the development of second-order ToM reasoning in our Turkish sample. Note that there were only 9 children (out of 41) in the younger group who scored more than 0 in the listening span task. However, for those 9 children, the listening span task score still significantly predicts the second-order false belief score in the ordinal logistic regression models. Further cross-linguistic studies are needed to unravel possible developmental differences in the listening span task between children speaking typologically different languages.

Now we will focus on the results about the role of syntactic recursion in the development of second-order ToM that we tested by constructing a second-order relative clause task (REL_2). As mentioned before, there is no consensus on the relationship between first-order relative clauses and first-order ToM [56–58]. In line with those studies that showed a positive relationship between the two, in the younger age group, we found a significant relationship between the second-order false belief judgment score and our syntactic recursion task, a relationship of *r*_*s*_ = .31 (*p =* .04) when age was controlled for. Moreover, we found a significant relationship between the second-order false belief justification score and our syntactic recursion task, *r*_*s*_ = .47 (*p <* .001) when age was controlled for. This two-fold positive relationship supports the view that a purely structural parallel between the linguistic realm and reasoning [43,54] does hold for the development of second-order ToM. However, this relationship completely disappeared when we controlled for the complex working memory task. This loss is due to the very strong correlation between the complex working memory task and the second-order relative clause task (*r*_*s*_ = .66, *p <* .001 for the younger group, and *r*_*s*_ = .41, *p =* .004 for the older group). This common variance of both tasks is again shared with the second-order false belief score. These strong mutual correlations are consistent with the hypothesis that the *serial processing bottleneck* seems to strongly affect all three tasks, at younger and older ages.

Our findings appear to be of interest to both language and memory researchers. The findings indicate, overall, that complex working memory strategies play a larger role in second-order ToM reasoning than syntactic recursion. However, given the strong overlap between the second-order relative clause task and the complex working memory task in both age groups, as shown in Table 5, it seems plausible that they both require similar complex working memory strategies that also facilitate second-order false belief reasoning. This similarity may give a hint at a possible convergence between the language and the memory explanations. As for the language explanation, hierarchical, syntactic embedding may be just the right representational tool to aid in the serialization process. The propositions are lexically selected by matrix verbs (e.g., “say” or “think”) as in embedded complement clauses (e.g., “John said *that Mary said that there was a flea in her cereal*. But in fact, she said *that there was a spider in her cereal*”), or dependent on a head noun (e.g., “the sheep”) as in embedded relative clauses (e.g., “Show me the sheep *that is pushing a monkey that is pushing a sheep*”). Furthermore, they are clearly demarcated and introduced by functional heads (“think *that*”; “the sheep *that*”). Thus, they are delivered in proper chunks ready for serializing them and passing smoothly through the *serial processing bottleneck* where central processes of interpretation take place. It is this chunking that may facilitate reasoning about the various beliefs (John’s and Mary’s, as pointed out above) in correct order. Importantly, horizontal, serial order directly follows from vertical, hierarchical structure: What is higher in the structural representation precedes in the linear string. As Hollebrandse and Roeper [55] state: “Recursion in grammar involves a translation between a hierarchical into a linear structure”.

The fact that the complex working memory task and not the second-order relative clause task is a better predictor for the second-order false belief reasoning may be due to the additional reasoning component in both our complex working memory task and the second-order false belief task, which is lacking in our second-order relative clause task. For example, to be able to pass the second-order relative clause task, one should parse the question “In which picture is there a sheep that is pushing a monkey that is pushing a sheep?” to obtain the meaning and select the proper picture that is the correct answer. Because the question includes second-order recursion, it also needs serial processing of information. However, it is possible to check the intermediate steps of the embedded parts of the sentence visually while parsing the sentence from the presented figure (Fig 1), which reduces the demands of working memory.

On the other hand, when answering the second-order false belief question “Where does Mary think that John will look for the chocolate?”, one is not only parsing the sentence to get the meaning, one also reasons about the question to come up with an answer. To be able to give a correct answer, one has to reason about the contradictory knowledge of Mary and John based on the reasoning rules, such as “Mary did not see John saw her hiding the chocolate, so she thinks that John thinks that the chocolate is still where he put it before, which is in the drawer, and therefore Mary thinks that John thinks that the chocolate in the drawer”. To achieve this reasoning, one should have efficient working memory strategies to overcome the *serial processing bottleneck*. Similarly, LST also requires an additional reasoning component beyond just holding in mind the to-be-remembered items and parsing the sentence, which is judging the truth value of the sentence. In addition, in our Turkish version of the LST, participants had to suppress the negative morphological marker of the verb when the truth value was negative, as discussed above.

As mentioned before, de Villiers et al. [27] argued that the truth contrasts in contexts with first-order complement clauses (“Mary said *that there was a spider in her cereal*. But it was just a raisin”) open the door for children to pass first-order false belief tasks and to recognize syntactic recursion. They further argue that, subsequently, understanding sentence recursion in contexts with second-order complement clauses allows children to pass higher-order theory of mind tasks (e.g., second-order false belief tasks). In addition to de Villiers et al.’s [27] argument that the truth contrasts might be an important stepping-stone in children’s understanding of sentence recursion which facilitates recursive false belief reasoning, we propose a general explanation for the development of children’s second-order false belief reasoning.

We surmise that children start to pass first-order false belief tasks when they learn to overcome the *serial processing bottleneck* by constructing more efficient reasoning rules to be able to attribute a false belief to another agent (first-order ToM) than applying the most salient reasoning strategy, that is, zero-order reasoning. Similarly, children pass second-order false belief tasks when they again learn to overcome the *serial processing bottleneck*, but this time by constructing efficient reasoning rules for second-order ToM reasoning. Our theory can be tested by adapting the standard second-order false belief tasks so that it is possible to derive children’s level of reasoning (i.e., zero-order, first-order, second-order) from their answers to the second-order ToM questions (see [53] for an example of a ‘Bake Sale’ story, in which a child can answer a second-order question with reference to three different objects, which correspond one-to-one to the three levels of ToM reasoning). We expect that children around the ages of 5 and 6 who cannot pass second-order ToM tasks will give mostly first-order answers instead of zero-order answers (reality bias). The *serial processing bottleneck* hypothesis also provides a procedural explanation of de Villiers et al.’s [27] following argument about children’s failure in second-order recursive structures (i.e., “Mary *believes* that John *thinks* that …”): “In both complementation and false belief reasoning, children first treat 2-level embedding as 1-level of structure. It is as if one piece of the hierarchy is flattened, or skipped over in parsing.” (p. 239).

We may generalize children’s failures at first-order and second-order false belief reasoning by saying that children’s incorrect answers are typically one order below the target order of false belief reasoning. Consistent with Miller’s account in terms of complexity (2009, p. 751), our parallel construal of first- and second-order ToM reasoning as well as the similarity in the patterns of failure may indicate that there is a common process underlying the development of first- and second-order ToM reasoning. If cognitive control over competing representations is gained and the nested structure of these representations can be serialized appropriately, children are capable of second-order ToM reasoning. Although we have discussed our results in terms of the complexity account, our results do not exclude the possibility that children’s recursive language abilities and complex working memory strategies may also contribute to a possible conceptual change that beliefs can be recursive.

Also note that we presented the tasks in the following fixed order: 1) simple working memory task; 2) second-order false belief task; 3) second-order relative clause task; 4) complex working memory task. Although there is no a priori reason that this particular presentation of the tasks might have produced the particular effects (for a similar case, see [74]), future research is needed to rule out any effect of the order of the tasks.

## Conclusions and Future Directions

As we predicted in the Subsection “Predictions” of the Introduction, there is a significant relation between the complex working memory task and the second-order false belief task and this relation is stronger than the relation between the simple working memory task and the second-order false belief task. Moreover, as we predicted, younger children’s (4–6) double-embedded relative task score is significantly correlated with their second-order false belief task score. However, our study shows that the main predictor of the development of second-order theory of mind (ToM) is the complex working memory task for both children’s judgment and justification answers for the second-order false belief question. Our study also shows that syntactic recursion and complex working memory measures are inter-related, suggesting common underlying capacities and processes. Based on these results, we propose that children’s second-order ToM develops when they are able to apply efficient reasoning rules to process embedded beliefs serially, thus overcoming the *serial processing bottleneck*.

To further test the *serial processing bottleneck* hypothesis, future research is needed, possibly with a training study in which the children are trained with a complex working memory span task while a simple working memory task is used in a control group. In this way, the effect of complex working memory strategies on second-order ToM reasoning can be observed. Moreover, to test whether children’s second-order false belief reasoning is supported more by second-order complement tasks, as argued by de Villiers et al. [27], or by complex memory tasks, one could also design a training study in which children on the brink of second-order ToM are subjected to training regimes consisting of second-order ‘memory of complement’ tasks (condition 1) or various complex working memory tasks (condition 2) and compare their improvements on second-order false belief tasks. Furthermore, to test whether the relationship between syntactic recursion and second-order false belief reasoning holds exclusively for recursion on the clause level or for recursion of any constituent, possessive recursions (as in “Mary’s friend’s dress”) might be used [75]. In addition to testing these hypotheses with behavioral data, constructing computational cognitive models by using cognitive architectures is a promising line of research (e.g., [31]).

## Supporting Information

S1 Materials (PDF)
