# Supplementary material for: Syntactic Recursion Facilitates and Working Memory Predicts Recursive Theory of Mind
Source: PLoS One. 2017 Jan 10;12(1):e0169510. doi: 10.1371/journal.pone.0169510 (PMC5225003; doi:10.1371/journal.pone.0169510)
Supplement: S1 Materials — (PDF) [file pone.0169510.s001.pdf]

Second-order Relative Clause Task (REL_2) Questions and Schematic Figures

S1 REL_2 Practice Question: In which picture is there a rabbit that is tickling a mouse that is tickling a rabbit? (“Hangi resimde tavşanı gıdıklayan fareyi gıdıklayan bir tavşan var?”)

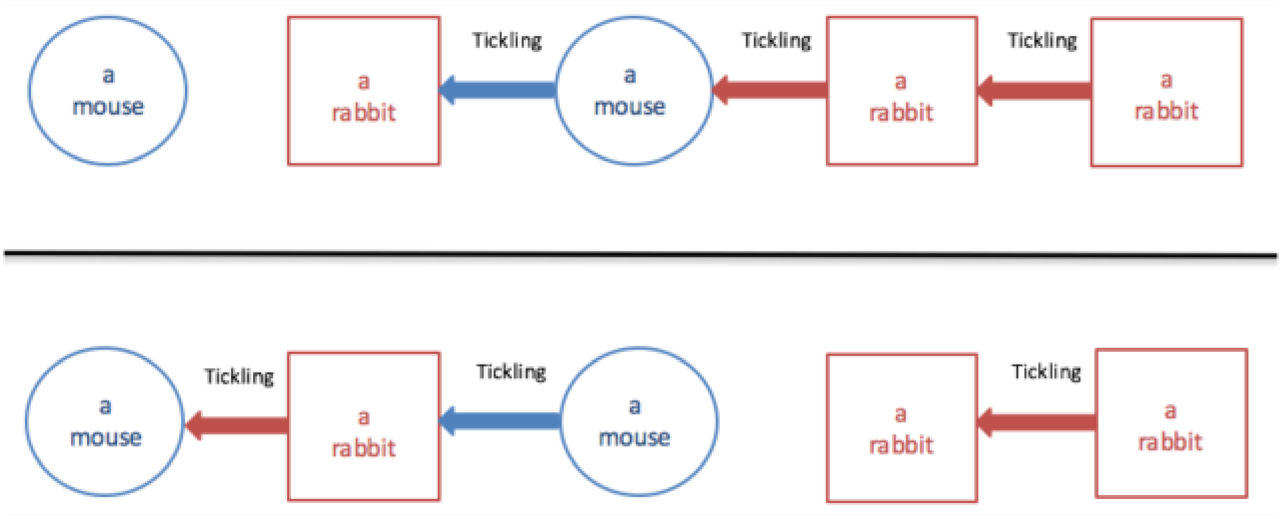

S1 REL_2 Lion-Gorilla: In which picture is there a lion that is biting a lion that is biting a gorilla? (“Hangi resimde gorili ısıran aslanı ısıran bir aslan var?”)

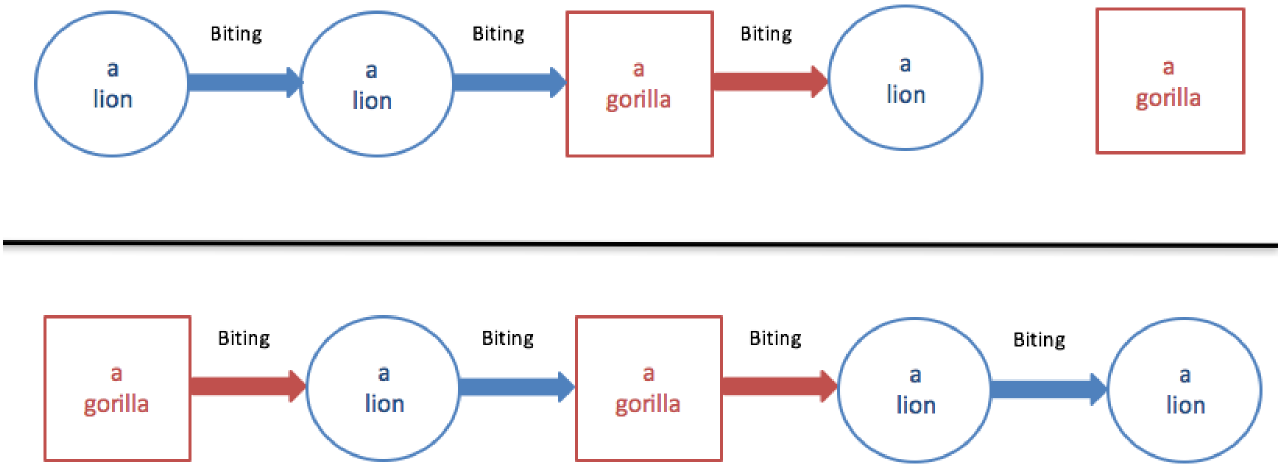

S1 REL_2 Horse-Camel: In which picture is there a horse that is caressing a camel that is caressing a horse? ("Hangi resimde atı okşayan deveyi okşayan bir at var?")

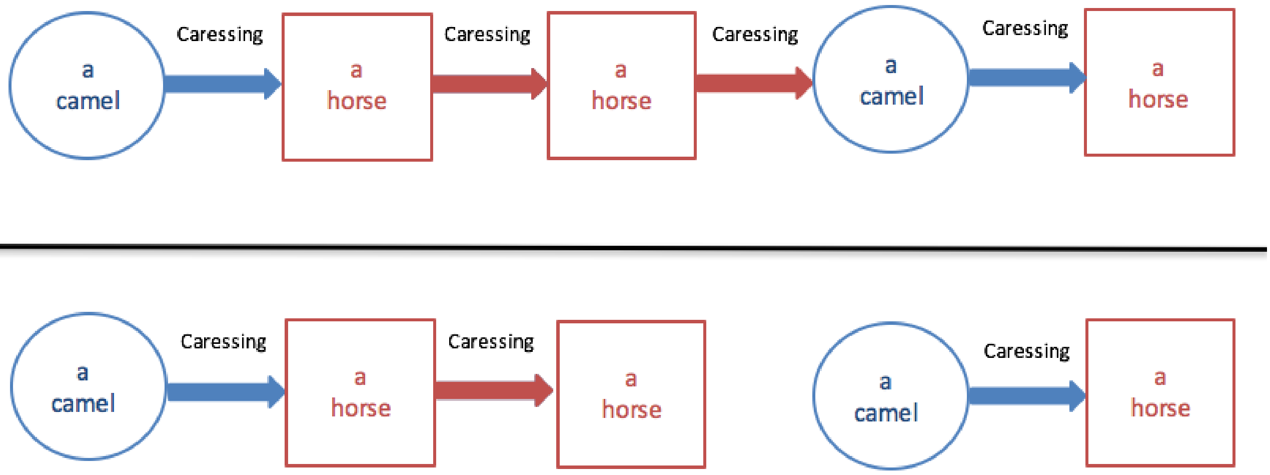

S1 REL_2 Mouse-Rabbit: In which picture is there a mouse that is kissing a rabbit that is kissing a
mouse? ("Hangi resimde fareyi öpen tavşanı open bir fare var?")

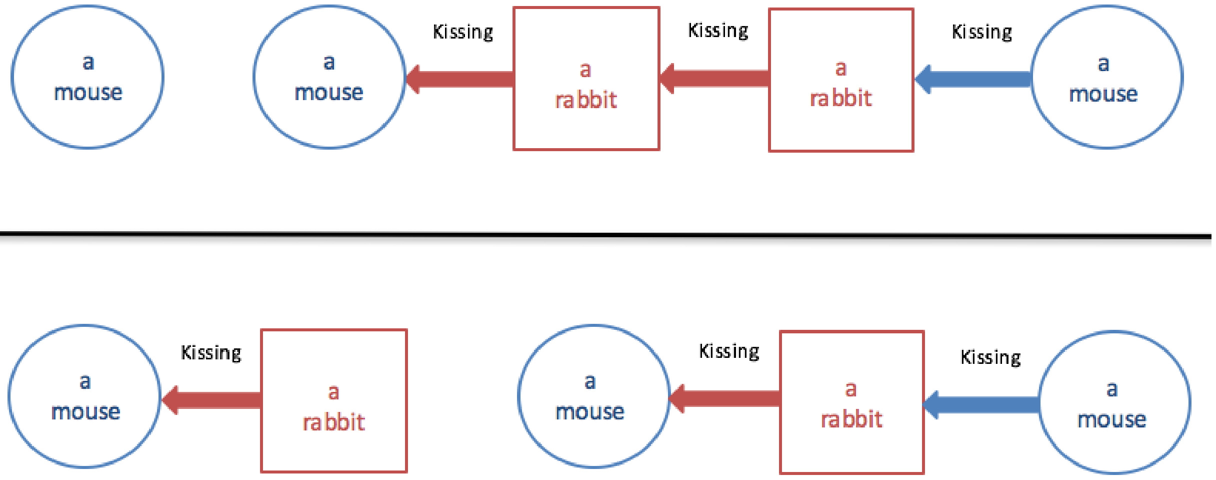

S1 REL_2 Cat-Dog: In which picture is there a dog that is licking a cat that is licking a dog? (“Hangi
resimde köpeği yalayan kediyi yalan bir kopek var?”)

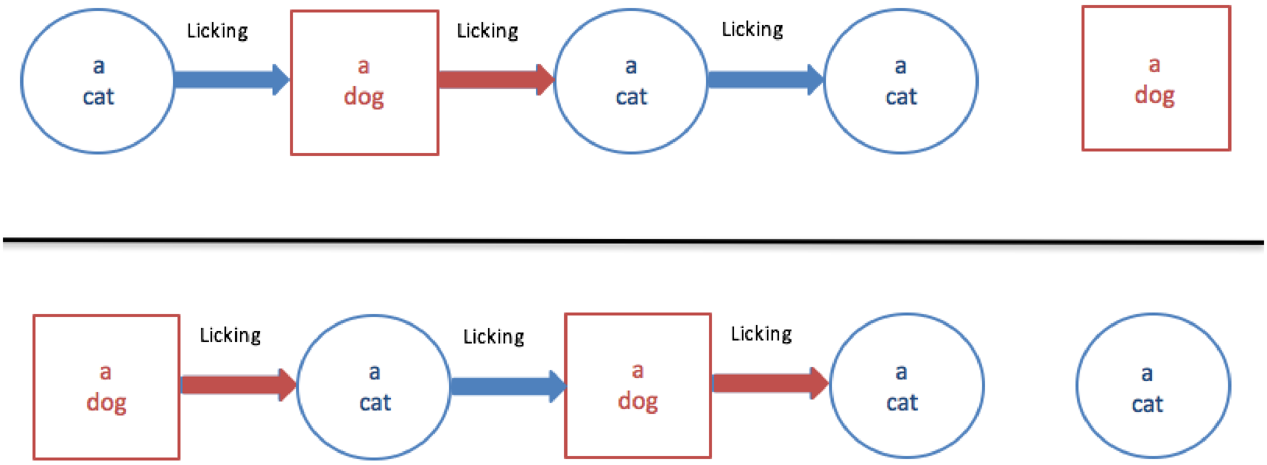

S1 REL_2 Sheep-Monkey: In which picture is there a sheep that is pushing a monkey that is pushing a sheep? (“Hangi resimde kuzuyu iten maymunu iten bir kuzu var?”)

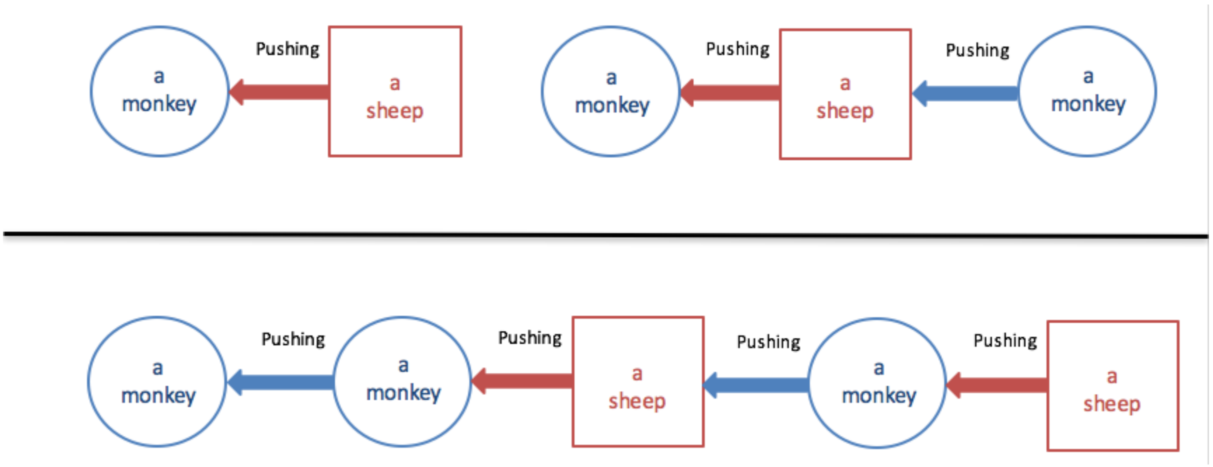

S1 REL_2 Goat-Cow: In which picture is there a goat that is nudging a cow that is caressing a goat?

(“Hangi resimde keçiyi okşayan ineği boynuzlayan bir keçi var?”)

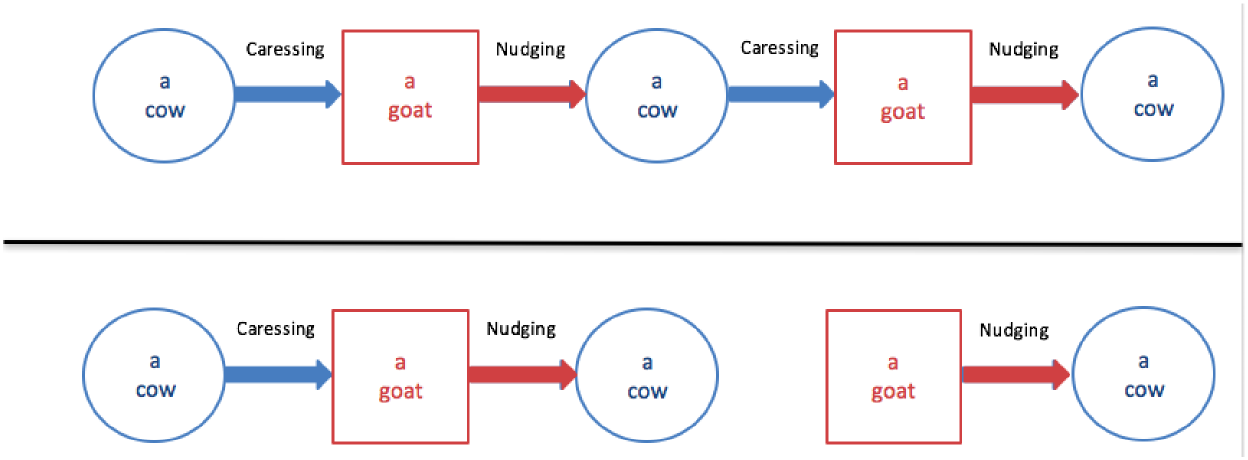

S1 Word Span Task Stimuli (WST)

<u>SETS OF 2</u>

Köşk – Muz
Pil – Üst
Buz – Dört

<u>SETS OF 3</u>

Göl - Saç - Tuz
Sev - Kürk - Bel
Kir - Ut – Pas

<u>SETS OF 4</u>

Kaş - Sos - Göc - Yat
Cam - But - Sal - Köy
Zar - Kuş - Tüm - Can

<u>SETS OF 5</u>

Suc - Kek - Böl - Top - Zam
Bal - Kurt - As - Tat - Cöp
Ot - Son - Türk - Seç – Kol

<u>SETS OF 6</u>

Hak - Sus - Tek - Mum - Dip - Kar
Kes - Bin - Ter - Aşk - Yut - Sel
Tren - Kel - Söz - An - Koy - Tez

<u>SETS OF 7</u>

Ak - Top - Su - Alt - Bey - Bol - Mart
Tel - Poz - At - Bil - Yok - Fes - Tür
Kış - Ver - Han - Bot - Yıl - Post - Kül

<u>SETS OF 8</u>

Tam - Bak - Uç - Göz - Hal - Boş - Ek - Yurt
Üç - Kas - Al - Mülk - Bir - Tut - Dil - Kum
Bul - Pek - On - Fal - Var - El - Ses - Genç

S1 Listening Span Task Stimuli

Used in the trials

1. Çocuklar okula gider.
2. Balıklar havada yaşar.
3. Ağaçlar dans eder.

SETS OF 2

1

1. Biber acıdır.
2. Kediler okulda çalışır.

2

1. Filler çok küçüktür.
2. Ayakkabı ayağa giyilir.

3

1. İnsanlar saçlıdır.
2. Çicekler fare kovalar.

4

1. Ayılar araba sürer.
2. Havuçlar turuncudur.

5

1. Gece karanlıktır.
2. Portakallar suda yaşar.

6

1. Ateş sıcaktır.
2. Balıklar konuşur.

SETS OF 3

1

1. Otobüslerle tatile gideriz.
2. Toplar karedir.
3. Öğretmenler ağaçta yetişir.

2

1. Muzlar bisiklete biner.
2. Elimiz beş parmaklıdır.
3. Soğan acıdır.

3

1. Otobüsler oyuncakla oynar.
2. Kuşlar kanatlıdır.
3. Elmalar ağaçta yetişir.

4

4. Piyanolar müzik çalar.
5. Kardeşlerimiz kuyrukludur.
6. Burnumuzla görürüz.

5

4. Ayağımız çenelidir.
5. Güneş sıcaktır.
6. Taşlar serttir.

6

4. Kaşıklarla yazı yazarız.
5. Limon sarıdır.
6. Köpekler kedileri kovalar.

SETS OF 4

1

1. Zürafalar uzun boyludur.
2. Çiçekler pasta sever.
3. Portakallar kulaklıdır.
4. Öğretmenler okulda çalışır.

2

1. Otobüsler konuşur.
2. Bankalardan para çekeriz.
3. Kışlar sıcaktır.
4. Pastalar tatlıdır.

3

1. Gökyüzü kırmızıdır.
2. Bebekler ağlar.
3. Köpekler konuşur.
4. Muzlar tatlıdır.

4

1. Armutlar mavidir.
2. Şapkalar başa giyilir.
3. Tavşanlar saati gösterir.
4. Filler büyüktür.

5

1. İnsanlar iki ayaklıdır.
2. Portakallar siyahtır.
3. Kediler futbol oynar.
4. Kitapları okuruz.

6

1. Tavşanlar ağaçta yetişir.
2. Biberler yeşildir.
3. Portakallar markette satılır.
4. İnsanlar üç gözlüdür.

SETS OF 5

1

1. Babalar kanatlıdır.
2. Dondurma soğuktur.
3. Portakallar gitar çalar.
4. Arabalar benzinle çalışır.
5. Fareler çok büyüktür.

2

1. Havuçlar mavidir.
2. Kulaklarımızla görürüz.
3. Portakallar turuncudur.
4. Tavuklar yumurta yapar.
5. Bıçak keskindir.

3

1. Elmalar pembedir.
2. Karıncalar yavaştır.
3. Dondurma sıcaktır.
4. Kediler fare kovalar.
5. Bebekler tüylüdür.

4

1. Kuşlar kocamandır.
2. Motorsikletler havlar.
3. Bıçaklar yumuşaktır.
4. Bulutlar beyazdır.
5. Tavuklar yazı yazar.

5

1. Gemiler uçar.
2. Kareler yuvarlaktır.
3. Çorabı ayağımıza giyeriz.
4. Bisikletler süt içer.
5. İnsanlar iki kulaklıdır.

6

1. Uçaklar kanatlıdır.
2. Elmalar şarkı söyler.
3. Dağlar çok küçüktür.
4. Sandalyeler ayaklıdır.
5. Makaslar kağıt keser.

SETS OF 6

1

1. Muzlar dişlidir.
2. Köpekler gitar çalar.
3. Bacağımız parmaklıdır.
4. Mektupları pulla göndeririz.
5. Muzlar sarıdır.
6. Kurbağalar zıplar.

2

1. Oyuncak ayılar yumuşaktır.
2. Ördekler suda yaşar.
3. Çocuklar üç kolludur.
4. Evimiz şarkı söyler.
5. Ördekler beş ayaklıdır.
6. Kar soğuktur.

3

1. Saatler zamanı gösterir.
2. Ayran tatlıdır.
3. Kurbağalar uzun kulaklıdır.
4. Ağaçlar müzik çalar.
5. Toplar yuvarlaktır.
6. Balıklar suda yaşar.

4

1. Arılar sokar.
2. Koyunlar kuyrukludur.
3. İnekler uçar.
4. Köpek balığıkocamandır.
5. Bulutlar siyahtır.
6. Pamuk ağırdır.

5

1. Ağaçlar tüylüdür.
2. Marketler yiyecek satar.

3. Domates kırmızıdır.
4. Kediler çok büyüktür.
5. Tavşanlar uzun kulaklıdır.
6. Tavuklar okula gider.

6

1. Kirazlar mavidir.
2. Ağaçlar yapraklıdır.
3. Demir hafiftir.
4. Yılanlar zıplar.
5. Kekler tatlıdır.
6. Tekerlekler karedir.

S1 Three versions of Birthday Puppy and Chocolate Stories with their drawings

Birthday Puppy Story (Neutral)

Bugün Mehmet'in doğum günü ve annesi ona yavru bir köpekle sürpriz yapmak istiyor. Mehmet'in annesi yavru köpeği bodruma saklıyor. Mehmet annesine, "Anneciğim, doğum günüm için bana yavru bir köpek almanı çok istiyorum" diyor. Annesinin yavru köpekle Mehmet'e sürpriz yapmak istediğini unutma! Bu yüzden ona yavru bir köpek aldığını söylemek yerine annesi, "Üzgünüm Mehmetciğim, doğum günün için sana yavru bir köpek almadım. Onun yerine sana çok güzel bir oyuncak aldım" diyor.

Reality control question: **Annesi doğum günü için Mehmet'e gerçekten ne aldı?**

Şimdi Mehmet annesine "Dışarıya oynamaya çıkıyorum." diyor. Dışarıya çıkarken patenlerini almak için bodruma iniyor. Bodrumda doğum günü hediyesi yavru köpeği buluyor! Kendi kendine "Vay canına, annem bana oyuncak almamış, gerçekten doğum günüm için bana yavru bir köpek almış" diyor. Annesi Mehmet'in bodruma indiğini ve doğum günü hediyesi yavru köpeği bulduğunu görmüyor.

1st order ignorance: **Mehmet doğum günü için annesinin ona yavru bir köpek aldığını biliyor mu?**

Linguistic control: **Annesi Mehmet'in bodrumdaki doğum günü hediyesi yavru köpeği gördüğünü biliyor mu?**

O sırada zır zır zır zır telefon çalıyor! Mehmet'in anneannesi doğum günü partisinin saat kaçta olduğunu öğrenmek için arıyor. Anneannesi telefonda Mehmet'in annesine "Mehmet doğum günü için ona gerçekten ne aldığını biliyor mu?" diye soruyor. Şimdi hatırlayalım, Mehmet'in annesi, doğum günü için Mehmet'e aldığı şeyi Mehmet'in gördüğünü bilmiyor.Daha sonra anneanne Mehmet'in annesine "Mehmet doğum günü için ona ne aldığını düşünüyor?" diye soruyor.

2nd order false belief: **Mehmet'in annesi anneanneye ne cevap verir?**
Justification: **Mehmet'in annesi neden böyle bir cevap verir?**

Birthday Puppy Story (-DI)

Dün Mehmetlerdeydim. Mehmet'in doğum günüydü ve annesi ona yavru bir köpekle sürpriz yapmak istedi. Mehmet'in annesi yavru köpeği bodruma sakladı.Mehmet annesine, "Anneciğim, doğum günüm için bana yavru bir köpek almanı çok istiyorum" dedi. Annesinin yavru köpekle Mehmet'e sürpriz yapmak istediğini

unutma! Bu yüzden ona yavru bir köpek aldığını söylemek yerine annesi, “Üzgünüm Mehmetciğim, doğum günün için sana yavru bir köpek almadım. Onun yerine sana çok güzel bir oyuncak aldım” dedi.

***Reality control question*: Annesi doğum günü için Mehmet’e gerçekten ne aldı?**

Mehmet annesine “Dışarıya oynamaya çıkıyorum.” dedi. Dışarıya çıkarken patenlerini almak için bodruma indi. Bodrumda doğum günü hediyesi yavru köpeği buldu! Kendi kendine “Vay canına, annem bana oyuncak almamış, gerçekten doğum günüm için bana yavru bir köpek almış” dedi. Annesi Mehmet’in bodruma indiğini ve doğum günü hediyesi yavru köpeği bulduğunu görmedi.

***1st order ignorance:* Mehmet doğum günü için annesinin ona yavru bir köpek aldığını biliyor muydu?**
***Linguistic control:* Annesi Mehmet’in bodrumdaki doğum günü hediyesi yavru köpeği gördüğünü biliyor muydu?**

O sırada zır zır zır zır telefon çaldı! Mehmet’in anneannesi doğum günü partisinin saat kaçta olduğunu öğrenmek için aradı.Anneannesi telefonda Mehmet’in annesine “Mehmet doğum günü için ona gerçekten ne aldığını biliyor mu?” diye sordu. Şimdi hatırlayalım, Mehmet’in annesi, doğum günü için Mehmet’e aldığı şeyi Mehmet’in gördüğünü bilmiyordu. Daha sonra anneanne Mehmet’in annesine “Mehmet doğum günü için ona ne aldığını düşünüyor?” diye sordu.

***2nd order false belief:* Mehmet’in annesi anneanneye ne cevap verdi?**
***Justification:* Mehmet’in annesi neden böyle bir cevap verdi?**

Birthday Puppy Story (-MIŞ)

Bak Mehmet. Geçen hafta Mehmet’in doğum günüymüş. Annesi ona yavru bir köpekle sürpriz yapmak istemiş. Mehmet’in annesi yavru köpeği bodruma saklamış. Mehmet annesine, “Anneciğim, doğum günüm için bana yavru bir köpek almanı çok istiyorum” demiş. Annesinin yavru köpekle Mehmet’e sürpriz yapmak istediğini unutma! Bu yüzden ona yavru bir köpek aldığını söylemek yerine annesi, “Üzgünüm Mehmetciğim, doğum günün için sana yavru bir köpek almadım. Onun yerine sana çok güzel bir oyuncak aldım” demiş.

***Reality control question*: Annesi doğum günü için Mehmet’e gerçekten ne almış?**

Mehmet annesine “Dışarıya oynamaya çıkıyorum.” demiş.Dışarıya çıkarken patenlerini almak için bodruma inmiş. Bodrumda doğum günü hediyesi yavru köpeği bulmuş! Kendi kendine “Vay canına, annem bana oyuncak almamış, gerçekten doğum günüm için bana yavru bir köpek almış” demiş. Annesi Mehmet’in bodruma indiğini ve doğum günü hediyesi yavru köpeği bulduğunu görmemiş.

1st order ignorance: **Mehmet doğum günü için annesinin ona yavru bir köpek aldığını biliyor muymuş?**
Linguistic control: **Annesi Mehmet'in bodrumdaki doğum günü hediyesi yavru köpeği gördüğünü biliyor muymuş?**

O sırada zır zır zır zır telefon çalmış! Mehmet'in anneannesi doğum günü partisinin saat kaçta olduğunu öğrenmek için aramış.Anneannesi telefonda Mehmet'in annesine "Mehmet doğum günü için ona gerçekten ne aldığını biliyor mu?" diye sormuş.

Şimdi hatırlayalım, Mehmet'in annesi, doğum günü için Mehmet'e aldığı şeyi Mehmet'in gördüğünü bilmiyormuş. Daha sonra anneanne Mehmet'in annesine "Mehmet doğum günü için ona ne aldığını düşünüyor?" diye sormuş.

2nd order false belief: **Mehmet'in annesi anneanneye ne cevap vermiş?**
Justification: **Mehmet'in annesi neden böyle bir cevap vermiş?**

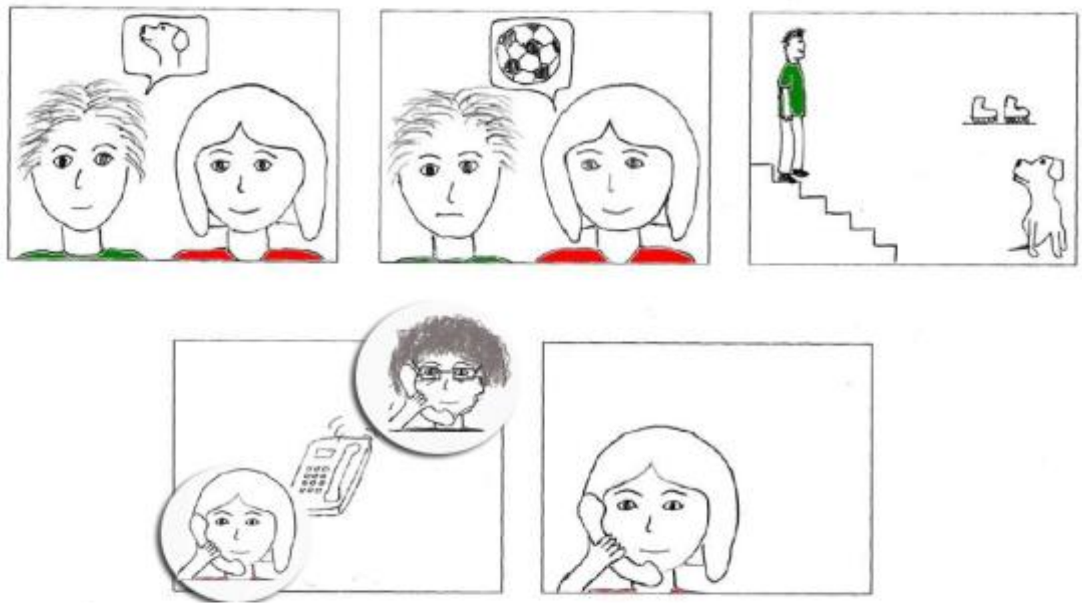

Adapted from Flobbe et al. [63] under a CC BY license, with permission from the authors. The grandmother image was added to the original drawing in order to make the story more explicit.

Chocolate Bar Story (NEUTRAL)

Bak, bunlar Can ile Ece kardeşler.Oturma odasında oynuyorlar.Biraz sonra anneleri alışverişten dönüyor, torbadan bir paket çikolata çıkarıyor. Çikolatayı Can'a veriyor.Ece'ye hiç çikolata vermiyor çünkü yaramazlık yapıyor. Can çikolatanın birazını yiyor ve kalanını çekmeceye koyuyor. Ece'ye hiç çikolata vermiyor. Ece da buna çok sinirleniyor. Can mutfağa annesine yardım etmek için bulaşıkları yıkamaya gidiyor. Ece oturma odasında tek başına oturuyor. Can ise mutfakta. Ece Can'a sinirlendiği için çikolatayı saklıyor. Çikolatayı çekmeceden alıyor ve oyuncak

sandığına koyuyor. Can bulaşıkları yıkamakla meşgul. Can, meyve kabuklarını bahçedeki çöp kovasına atmaya giderken pencereden oturma odasını görüyor. Ece'nin çikolatayı çekmeceden alıp oyuncak sandığına koyduğunu görüyor. Ece ise Can'ı görmüyor.

Reality control question: **Çikolata şimdi nerede?**
1st order ignorance: **Can, Ece'nin çikolatayı oyuncak sandığına sakladığını biliyor mu?**
Linguistic control: **Ece çikolatayı saklarken Can'ın onu gördüğünü biliyor mu?**

Can bulaşıkları bitiriyor. Karnı acıkıyor. Çikolatasından biraz yemek istiyor. Can oturma odasına giriyor. "Canım biraz çikolata istiyor." diyor.

2nd order false belief: **Ece çikolata için Can'ın nereye bakacağını düşünüyor?**
Justification: **Ece neden böyle düşünüyor?**

Chocolate Bar Story (-DI)

Bak, bunlar Can ile Ece kardeşler. Geçen gün onların evindeydim. Oturma odasında oynuyorlardı. Biraz sonra anneleri alışverişten döndü, torbadan bir paket çikolata çıkardı. Çikolatayı Can'a verdi. Ece'ye hiç çikolata vermedi çünkü yaramazlık yapıyordu. Can çikolatanın birazını yedi ve kalanını çekmeceye koydu. Ece'ye hiç çikolata vermedi. Ece da buna çok sinirlendi. Can mutfağa annesine yardım etmek için bulaşıkları yıkamaya gitti. Ece oturma odasında tek başına oturuyordu. Can ise mutfaktaydı. Ece Can'a sinirlendiği için çikolatayı sakladı. Çikolatayı çekmeceden aldı ve oyuncak sandığına koydu. Can bulaşıkları yıkamakla meşguldü. Can, meyve kabuklarını bahçedeki çöp kovasına atmaya giderken penceren oturma odasını görüyordu. Ece'nin çikolatayı çekmeceden alıp oyuncak sandığına koyduğunu gördü. Ece ise Can'ı görmedi.

Reality control question: **Çikolata neredeydi?**
1st order ignorance: **Can, Ece'nin çikolatayı oyuncak sandığına sakladığını biliyor muydu?**
Linguistic control: **Ece çikolatayı saklarken Can'ın onu gördüğünü biliyor muydu?**

Can bulaşıkları bitirdi. Karnı acıktı. Çikolatasından biraz yemek istedi. Can oturma odasına gitti. "Canım biraz çikolata istiyor." dedi.

***2nd order false belief*:** **Ece çikolata için Can'ın nereye bakacağını düşündü?**
***Justification*:** **Ece neden böyle düşündü?**

Chocolate Bar Story (-MIŞ)

Bak, bunlar Can ile Ece kardeşler. Geçenlerde Can ile Ece oturma odasında oynuyorlarmış. Biraz sonra anneleri alışverişten dönmüş, torbadan bir paket çikolata çıkarmış. Çikolatayı Can’a vermiş. Ece’ye hiç çikolata vermemiş çünkü yaramazlık yapıyormuş. Can çikolatanın birazını yemiş ve kalanını çekmeceye koymuş. Ece’ye hiç çikolata vermemiş. Ece da buna çok sinirlenmiş. Can mutfağa annesine yardım etmek için bulaşıkları yıkamaya gitmiş. Ece oturma odasında tek başına oturuyormuş. Can ise mutfaktaymış. Ece Can’a sinirlendiği için çikolatayı saklamış. Çikolatayı çekmeceden almış ve oyuncak sandığına koymuş. Can bulaşıkları yıkamakla meşgulmüş. Can, meyve kabuklarını bahçedeki çöp kovasına atmaya giderken pencereden oturma odasını görüyormuş. Ece’nin çikolatayı çekmeceden alıp oyuncak sandığına koyduğunu görmüş. Ece ise Can’ı görmemiş.

***Reality control question*: Çikolata neredeymiş?**
***1st order ignorance*: Can, Ece’nin çikolatayı oyuncak sandığına sakladığını biliyor muymuş?**
***Linguistic control*: Ece çikolatayı saklarken Can’ın onu gördüğünü biliyor muymuş?**

Can bulaşıkları bitirmiş. Karnı acıkmış. Çikolatasından biraz yemek istemiş. Can oturma odasına gitmiş. “Canım biraz çikolata istiyor.” demiş.

***2nd order false belief*: Ece çikolata için Can’ın nereye bakacağını düşünmüş?**
***Justification*: Ece neden böyle düşünmüş?**

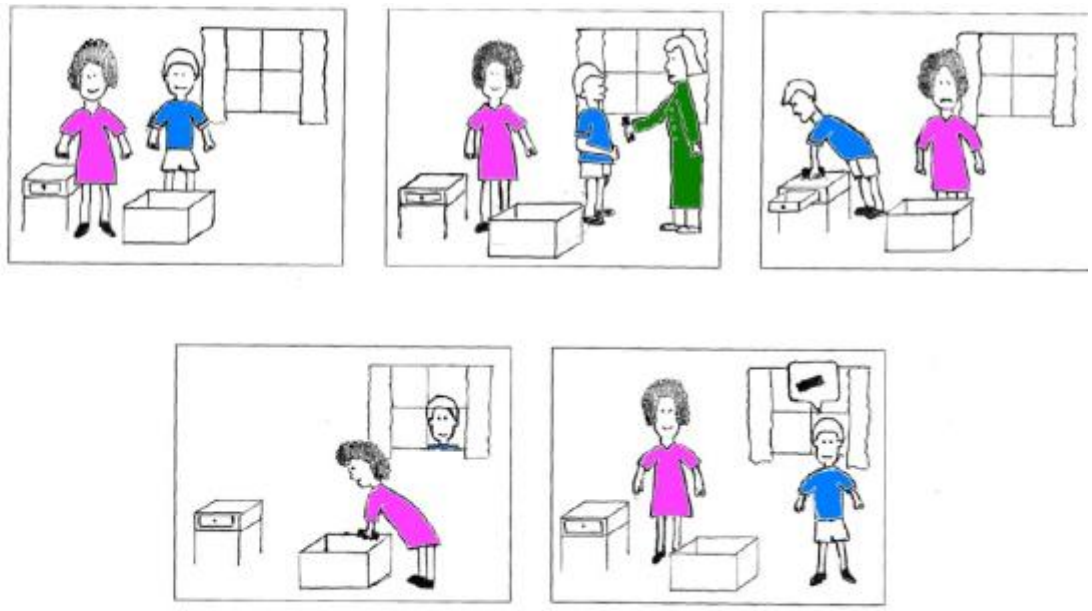

Adapted from Flobbe et al. [63] under a CC BY license, with permission from the authors.
